# Exacerbation History and Risk of Myocardial Infarction and Pulmonary Embolism in COPD

**DOI:** 10.1016/j.chest.2024.07.150

**Published:** 2024-07-31

**Authors:** Oskar Wallström, Caroline Stridsman, Anne Lindberg, Fredrik Nyberg, Lowie E.G.W. Vanfleteren

**Affiliations:** a COPD Center, Department of Respiratory Medicine and Allergology, Sahlgrenska University Hospital, Gothenburg, Sweden; b Department of Internal Medicine and Clinical Nutrition, Institute of Medicine, Sahlgrenska Academy, University of Gothenburg, Gothenburg, Sweden; c Department of Public Health and Clinical Medicine, The OLIN-unit, Umeå University, Umeå, Sweden; d School of Public Health and Community Medicine, Institute of Medicine, Sahlgrenska Academy, University of Gothenburg, Gothenburg, Sweden

**Keywords:** acute exacerbations of COPD, cardiovascular adverse events, comorbidity, myocardial infarction, pulmonary embolism, retrospective nationwide registry cohort

## Abstract

**Background:**

Acute exacerbations (AEs) of COPD are increasingly recognized as episodes of heightened risk of cardiovascular events. It is not known whether exacerbation history is differentially associated with future myocardial infarction (MI) or pulmonary embolism (PE).

**Research Question:**

Is the number and severity of AEs of COPD associated with increased risk of MI or PE in a real-life cohort of patients with COPD?

**Study Design and Methods:**

We identified a cohort of 66,422 patients (≥ 30 years of age) with a primary diagnosis of COPD in the Swedish National Airway Register from January 2014 to June 2022, with complete data on lung function. Patients were classified by moderate (prescription of oral corticosteroids) and severe (hospitalization) exacerbations the year before index date and were followed until December 2022 for hospitalization or death from MI or PE, corresponding to > 265,000 patient-years, with a maximum follow-up time of 9 years. Competing risk regression, according to the Fine-Gray model, was used to calculate subdistribution hazard ratios with 95% CIs.

**Results:**

Compared with no AEs of COPD in the baseline period, AE of COPD number and severity were associated with increased long-term risk of both MI and PE in a gradual fashion, ranging from a subdistribution hazard ratio of 1.10 (95% CI, 0.97-1.24) and 1.33 (95% CI, 1.11-1.60), respectively, for one moderate exacerbation, to 1.82 (95% CI, 1.36-2.44) and 2.62 (95% CI, 1.77-3.89), respectively, for two or more severe exacerbations. In a time-restricted follow-up sensitivity analysis, the associations were stronger during the first year of follow-up and diminished over time.

**Interpretation:**

The risk of MI and PE increased with the frequency and severity of AEs of COPD in this large, real-life cohort of patients with COPD.

FOR EDITORIAL COMMENT, SEE PAGE 1262

**Take-home Points**

**Study Question:** Is the number and severity of acute exacerbations of COPD associated with increased risk of myocardial infarction (MI) or pulmonary embolism (PE) in a real-life cohort of patients with COPD?

**Results:** Exacerbation number and severity was associated with the future long-term rate of both MI and PE in a gradual fashion.

**Interpretation:** The risk of MI and PE increased with the frequency and severity of acute exacerbation of COPD in this large, real-life cohort of patients with COPD.

Acute exacerbations (AEs) of COPD are important events in the natural course of COPD, associated with accelerated decline in lung function, increased morbidity, lower quality of life, and increased risk of future exacerbations and mortality.^1^, ^2^, ^3^, ^4^, ^5^, ^6^, ^7^, ^8^ The risk of having a new AE of COPD and the risk of mortality increase in a gradual fashion, based on the frequency and severity of prior exacerbations.^6^

In the presence of an AE of COPD, the risk of myocardial infarction (MI) and pulmonary embolism (PE) is greatly elevated; importantly, both of these diagnoses contribute to higher mortality among patients with COPD.^9^^,^^10^ The time-dependent risk of cardiovascular events in the period immediately after an AE of COPD has been studied.^11^, ^12^, ^13^, ^14^, ^15^ However, only some studies have specifically addressed the long-term risk of cardiovascular events related to the number and severity of exacerbations in the last year, which is the method the Global Initiative for Obstructive Lung Disease (GOLD) currently recommends to determine the risk of future exacerbations to guide treatment strategies.^16^, ^17^, ^18^, ^19^

Therefore, we conducted a register-based cohort study using the Swedish National Airway Register (SNAR),^20^ with the aim to investigate the association between exacerbation frequency and severity in the last year with future risk of MI and PE in patients with COPD.

## Study Design and Methods

### Study Population

The SNAR was initiated in 2013 and includes data on patients with obstructive lung diseases, from primary and secondary care, registered in > 1,000 clinics across Sweden.^20^ In this study, patients with a physician-assessed primary diagnosis of COPD registered in the SNAR from January 2014 to June 2022, and ≥ 30 years of age on the index date, were included. The choice of January 2014 as the start of the study period reflects the earliest possible date with SNAR data. The index date was defined as the latest health care visit with a registered FEV_1_ in the SNAR within the predefined period. This ensures that all participants start the follow-up with relevant recent data on this important covariate.

### Patient Characteristics and Definitions

**Figure 1:**
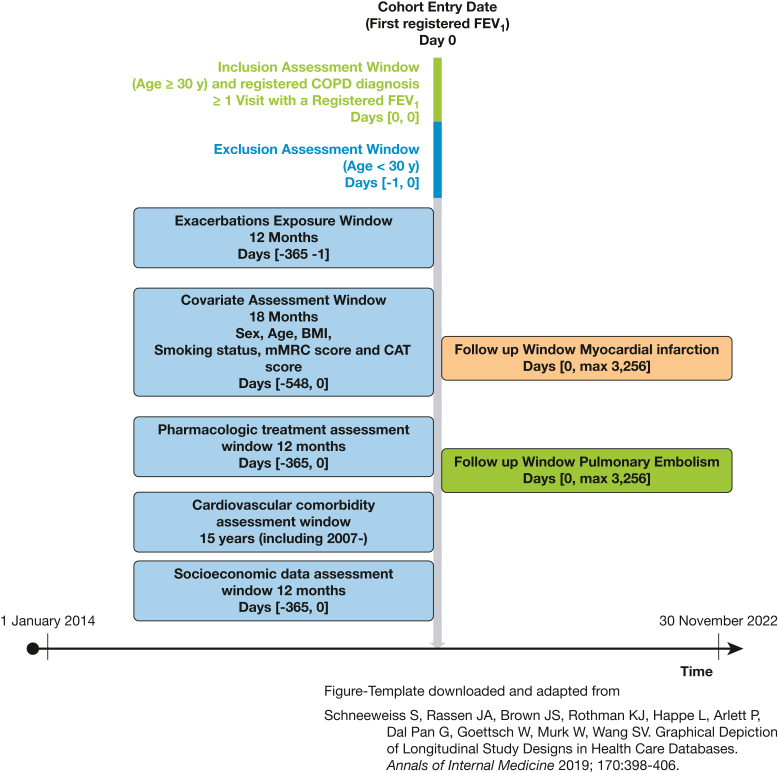
Cohort design, covariate assessment, and follow-up period. CAT = COPD Assessment Test; mMRC = modified Medical Research Council Dyspnea Scale.

Figure 1 provides an overview of the cohort covariate assessment in the different registers and follow-up periods. For covariates pertaining to cardiovascular comorbidities and outcome variables, we used data linkage between the SNAR, the National Patient Register (NPR), the National Cause of Death Register (NCDR), and the National Prescribed Drug Register (NPDR) to obtain relevant data.

To create exacerbation history strata, we used previously validated methods to identify moderate and severe exacerbations.^21^ Moderate exacerbations were defined as pharmacy prescriptions of short-term oral corticosteroids captured by Anatomical Therapeutic Chemical Classification System (ATC) code H02AB, retrieved from the NPDR. A list of corticosteroid treatments was manually reviewed to identify treatments prescribed for the COPD exacerbation indication. Severe exacerbations were defined as respiratory hospitalizations captured by International Classification of Diseases, 10th Revision (ICD-10) codes J41-J44, J96, J12-J18, or J20-J22 retrieved from NPR used as the main or secondary diagnosis in the electronic medical record.

**Figure 2:**
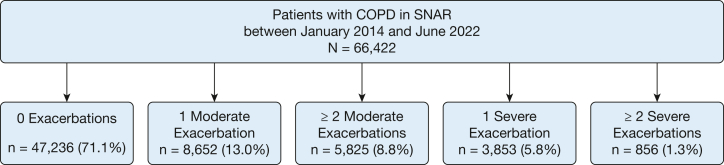
Flow diagram for distribution of exacerbation history groups. SNAR = Swedish National Airway Register.

In the main analysis, the exposure of patients with COPD was classified into five categories based on exacerbation history in the year prior to the index date: (1) no exacerbations; (2) one moderate exacerbation, no severe exacerbations; (3) two or more moderate exacerbations, no severe exacerbations; (4) one severe exacerbation; and (5) two or more severe exacerbations (Fig 2). In the case of exposure to both moderate and severe exacerbations, the classification was done on the highest severity meaning the exposure category was the number of severe exacerbations, similar to a previously developed method.^22^

### Primary Outcome and Follow-up

The primary outcomes were MI (ICD-10 I21-I22) and PE (ICD-10 I26), defined as the first of either (date of admission for) hospitalization (main or secondary diagnosis code from NPR) or (date of) out-of-hospital-death from a MI or PE (underlying cause of death from NCDR) during follow-up. Patients were followed from the index date for a maximum of 9 years to the occurrence of the outcome of interest (MI or PE), death from another cause, or the end of follow-up on November 30, 2022. The minimum amount of follow-up was 6 months for patients registered on June 1, 2022.

Sensitivity analyses were conducted, with the follow-up time restricted to 1, 2, and 5 years, respectively, for the main outcomes. The same competing risk regression model and covariates were used.

### Covariates

Covariates extracted from the SNAR included sex, age, BMI, smoking status and spirometry values (FEV_1_, FVC, and ratio FEV_1_/FVC), modified Medical Research Council Dyspnea Scale score, and COPD Assessment Test score. The latest registered datapoint in the period 18 months before the index date was used for smoking and BMI because such more stable covariates with more observations in the data set can be well accounted for in this way. Smoking status was categorized as patients who never smoked, patients who previously smoked (smoking-free for at least 6 months), and patients with active tobacco use. BMI was categorized as normal weight (18.5-24.9 kg/m^2^), underweight (< 18.5 kg/m^2^), overweight (25-29.9 kg/m^2^), and obese (> 30 kg/m^2^). Swedish reference values were used to calculate the percent predicted FEV_1_ and FVC.^23^^,^^24^ Lung function was divided into GOLD grades based on FEV_1_ % predicted: GOLD 1 (FEV_1_ ≥ 80%), GOLD 2 (≥ 50% FEV_1_ < 80%), GOLD 3 (≥ 30% FEV_1_ < 50%), and GOLD 4 (FEV_1_ < 30%).^16^

Maintenance COPD and cardiovascular pharmacologic treatment were retrieved from NPDR and defined as at least one dispensation within 1 year before the index date (see e-Tables 1 and 2 for the ATC codes and definitions). All COPD inhaler combination therapies were defined by dispensations of ATC codes for fixed single inhaler treatment.

The cardiovascular comorbidities selected as covariates were a previous history of MI (I21-I24), stroke (I61, I63), heart failure (I50), atrial fibrillation (I48), coronary artery disease (I20-I25), hypertension (I10 or I109), peripheral artery disease (I702, I739), diabetes type 2 (E11), and PE (I26), captured by ICD-10 codes as the main or concomitant diagnosis in NPR at any time prior to the index date but after January 1, 2007.

Socioeconomic status was based on the indicators education and income, obtained from the Longitudinal Integrated Database for Health Insurance and Labour Market Studies from the registered value in the year prior to the index date. Income was leveled into four quartiles based on the median salary in Sweden. Educational level was leveled into three categories: low (< 9 years), medium (9-12 years), and high (> 12 years).

### Ethical Approval

The study was approved by the Swedish Ethical Review Authority (No. 2019-04915).

### Statistical Analysis

We used descriptive statistics to present the baseline characteristics of the analyzed study participants. Normally distributed continuous values were described using mean ± SD. Categorical variables were described using counts and percentages.

**Figure 3:**
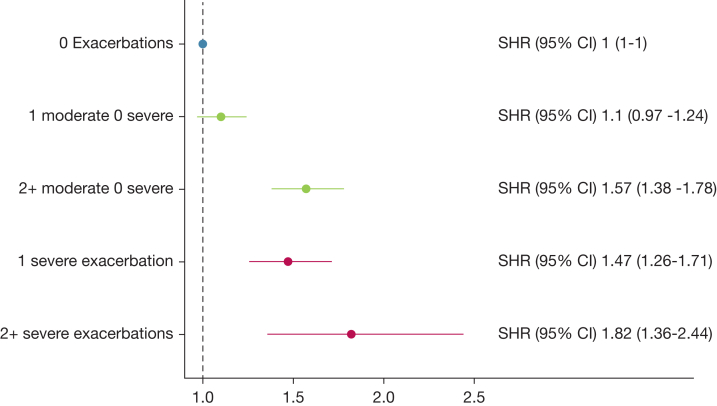
Forest plot of adjusted^a^ SHRs for myocardial infarction depending on baseline exacerbation history, from competing risk regression analysis for 66,422 Swedish patients diagnosed with COPD, registered in the Swedish National Airway Register, January 2014 to June 2022 and followed for up to 9 y. ^a^Adjusted for sex, age, BMI, smoking status, FEV_1_ % predicted, presence of baseline cardiovascular comorbidity, cardioprotective medications, and socioeconomic factors. SHR = subdistribution hazard ratio.

**Figure 4:**
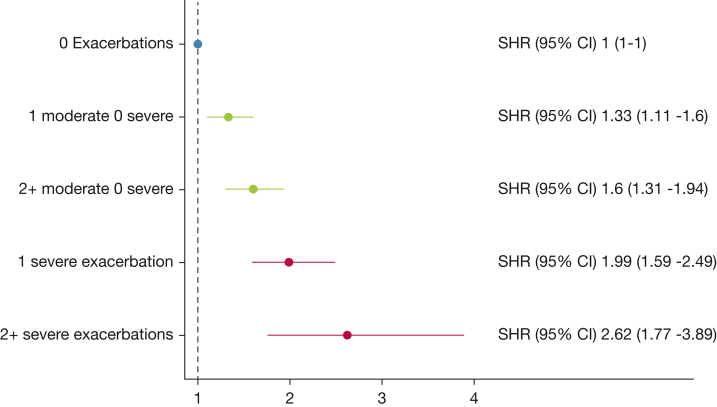
Forest plot of adjusted^a^ SHRs for pulmonary embolism depending on baseline exacerbation history, from competing risk regression analysis for 66,422 Swedish patients diagnosed with COPD, registered in the Swedish National Airway Register, January 2014 to June 2022 and followed for up to 9 years. ^a^Adjusted for sex, age, BMI, smoking status, FEV_1_ % predicted, presence of baseline cardiovascular comorbidity, cardioprotective medications, and socioeconomic factors. SHR = subdistribution hazard ratio.

**e-Figure 1:**
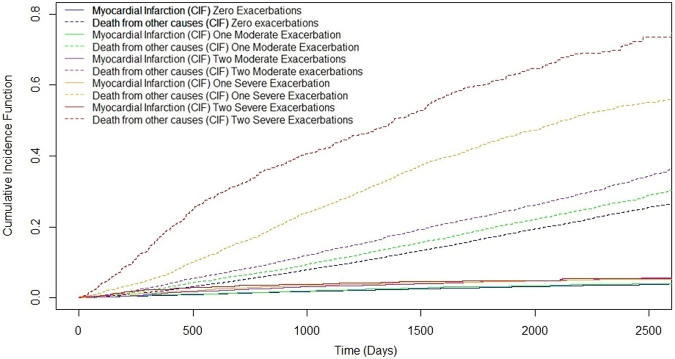
Cumulative incidence functions plot for the outcome of myocardial infarction with death from other causes as a competing risk, in 66422 Patients with COPD divided by baseline exacerbation history

**e-Figure 2:**
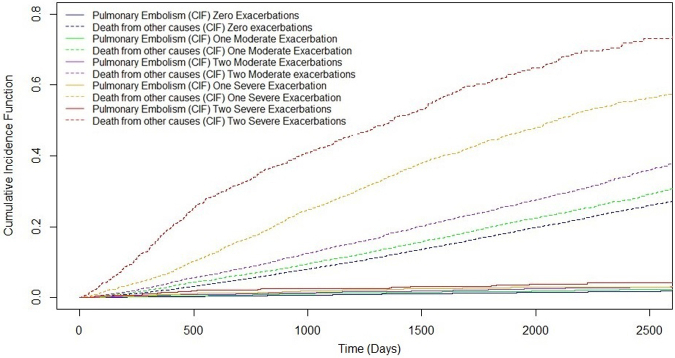
Cumulative incidence functions plot for the outcome of pulmonary embolism with death from other causes as a competing risk, in 66422 Patients with COPD divided by baseline exacerbation history

Competing risk regression, specifically the Fine-Gray proportional hazards model for subdistribution, was used to estimate subdistribution hazard ratios (SHRs) for the occurrence of MI and PE among patients with COPD, classified by their exacerbation history (Figs 3, 4). In this model, death from all other causes was considered as a competing risk.^25^ Crude and adjusted SHRs with 95% CIs were computed for MI and PE, with the group with no exacerbations in the year prior to the index date as the reference (e-Tables 3, 4). We adjusted both models for sex, age, BMI, smoking status, FEV_1_ % predicted at baseline, any previous cardiovascular comorbidity, cardioprotective medications, income, and educational level. Cumulative incidence function curves were constructed to complement our regression analysis in accordance with the recommendations for reporting Fine-Gray competing regression analyses (e-Figs 1, 2).^26^

The choice of covariates was made on their clinical relevance but also on their availability within our data sets. Of note, we did not adjust the model for COPD Assessment Test score or modified Medical Research Council Dyspnea Scale because of limited data on these variables.

To handle the small percentage of missing data on BMI (2.2%) and smoking (6.7%), we used multiple imputations by chained equations to impute the missing data on smoking and BMI for the main analysis to avoid introducing selection bias into the results. To account for the potential of misclassified asthma among patients who never smoked in the cohort of patients with COPD, we conducted a sensitivity analysis where we excluded patients who never smoked. We also conducted another sensitivity analysis including sleep apnea as a covariate in the competing risk regression model, treating missing on this variable as a factor level. Results from these sensitivity analyses, one including only patients with active tobacco use or who previously smoked and one adding sleep apnea as a covariate, are presented in e-Tables 5, 6.

The baseline data were defined using SAS version 15.2 (SAS Institute), and R version 4.3.1 (R Foundation for Statistical Computing) was used for further data management. The mice-package (version 3.16.0) was used for imputation, and the cmprsk-package (version 2.2-11) was used for the competing risk regression analyses according to the Fine-Gray model.

## Results

### Characteristics of the Study Population

Figure 2 shows a flow diagram of the study population. In total, 66,422 unique patients with COPD > 30 years of age on the index date with data on FEV_1_ were included in the cohort.

The baseline characteristics of the cohort are presented in Table 1, Table 2, Table 3. Mean FEV_1_ percent predicted was 60.3, 37.6% were patients with active tobacco use, 77.2% used bronchodilation therapy, and 50.2% were on inhaled corticosteroids (ICSs). Although 71.1% had no AEs of COPD in the year before the index date, 13.0% had one moderate, 8.8% had two or more moderate, 5.8% had one severe, and 1.3% had two or more severe AEs of COPD. With increasing frequency and severity of AEs of COPD, patients more often were female, were older, were underweight, and had a history of smoking. They also had lower lung function, were more frequently on combination treatment with ICSs, long-acting beta-2 agonist (LABA), and long-acting antimuscarinic agent (LAMA) (ie, triple therapy), and had a higher prevalence of cardiovascular comorbidity at baseline.

**Table 1:** Baseline Characteristics

Characteristic	Total Study Population	No. of Exacerbations, Moderate/Severe				
		0/0	1/0	≥ 2/0	0/1	0/≥ 2
Patients	66,422 (100)	47,236 (71.1)	8,652 (13.0)	5,825 (8.8)	3,853 (5.8)	856 (1.3)
Male sex	29,180 (43.9)	21,546 (45.6)	3,439 (39.7)	2,250 (38.6)	1,614 (41.9)	331 (38.7)
Age, y	70.3 ± 9.4	70.0 ± 9.4	69.7 ± 9.5	70.9 ± 9.1	73.2 ± 8.9	74.1 ± 8.3
BMI, kg/m^2^	26.9 ± 5.7	26.8 ± 5.3	27.1 ± 5.8	27.2 ± 5.7	26.3 ± 6.5	25.8 ± 6.8
BMI category^a^						
Obese	16,187 (24.4)	11,309 (23.9)	2,254 (26.1)	1,520 (26.1)	902 (23.4)	202 (23.6)
Normal weight	22,992 (34.6)	16,438 (34.8)	2,883 (33.3)	1,909 (32.8)	1,454 (37.7)	308 (36.0)
Underweight	3,473 (5.2)	2,271 (4.8)	453 (5.2)	251 (4.3)	377 (9.8)	121 (14.1)
Overweight	22,276 (33.5)	16,175 (34.2)	2,830 (32.7)	1,996 (34.3)	1,062 (27.6)	213 (24.9)
Smoking status^a^						
Active tobacco use	24,963 (37.6)	18,403 (39.0)	3,169 (36.6)	1,718 (29.5)	1,394 (36.2)	279 (32.6)
Previously smoked	29,763 (44.8)	20,444 (43.3)	3,947 (45.6)	3,016 (51.8)	1,900 (49.3)	456 (53.3)
Never smoked	7,225 (10.9)	5,244 (11.1)	939 (10.9)	664 (11.4)	319 (8.3)	59 (6.9)
Spirometry values						
FEV_1_	1.7 ± 0.7	1.8 ± 0.7	1.6 ± 0.7	1.5 ± 0.6	1.3 ± 0.6	1.0 ± 0.5
FEV_1_ % predicted	60.3 ± 18.5	62.4 ± 17.8	58.6 ± 18.6	55.9 ± 18.5	49.4 ± 18.6	41.0 ± 17.7
FVC	2.8 ± 0.9	2.9 ± 0.9	2.7 ± 0.9	2.6 ± 0.9	2.4 ± 0.8	2.1 ± 0.8
FVC % predicted	76.2 ± 17.5	77.4 ± 17.2	75.1 ± 17.3	73.3 ± 17.3	69.5 ± 18.8	64.2 ± 18.9
FEV_1_/FVC	0.6 ± 0.2	0.6 ± 0.1	0.6 ± 0.2	0.6 ± 0.2	0.6 ± 0.2	0.5 ± 0.2
GOLD grade						
1	9,721 (14.6)	7,731 (16.4)	1,116 (12.9)	587 (10.1)	257 (6.7)	30 (3.5)
2	37,801 (56.9)	28,326 (60.0)	4,733 (54.7)	3,024 (51.9)	1,524 (39.6)	194 (22.7)
3	15,574 (23.4)	9,597 (20.3)	2,305 (26.6)	1,775 (30.5)	1,519 (39.4)	378 (44.2)
4	3,326 (5.0)	1,582 (3.3)	498 (5.8)	439 (7.5)	553 (14.4)	254 (29.7)
Symptomatic burden						
CAT score	13.4 ±7.1	12.5 ± 6.6	14.5 ± 7.4	15.5 ± 7.8	16.3 ± 7.8	19.4 ± 7.9
mMRC score	1.6 ± 1.2	1.4 ± 1.1	1.8 ± 1.2	2.0 ± 1.2	2.4 ± 1.2	3.0 ± 1.1
GOLD classes A, B, and E						
A	14,800 (29.0)	12,962 (36.2)	1,838 (27.4)	0 (0.0)	0 (0.0)	0 (0.0)
B	27,725 (54.4)	22,846 (63.8)	4,879 (72.6)	0 (0.0)	0 (0.0)	0 (0.0)
E	8,458 (16.6)	0 (0.0)	0 (0.0)	4,583 (100.0)	3,152 (100)	723 (100.0)

Values are No. (%) or mean ± SD. CAT = COPD Assessment Test; GOLD = Global Initiative for Chronic Obstructive Lung Disease; mMRC = modified Medical Research Council Dyspnea Scale.

a BMI available for 64,928 patients, smoking status available for 61,951 patients, CAT score available for 46,214 patients, mMRC score available for 31,970 patients, and GOLD classification A to E available for 50,983 patients.

**Table 2:** Primary End Points and Cardiovascular Comorbidity at Baseline

Cardiovascular Event Outcomes and Baseline Comorbidities	Total Study Population (N = 66,422)	No. of Exacerbations, Moderate/Severe				
		0/0 (n = 47,236)	1/0 (n = 8,652)	≥ 2/0 (n = 5,825)	0/1 (n = 3,853)	0/≥ 2 (n = 856)
Myocardial infarction						
Person-years	26,5849	190,480	35,876	23,565	13,462	2,466
No. of events	2,260	1,434	291	289	197	49
Events/100,000 person-years	850	753	811	1,226	1,463	1,987
Pulmonary embolism						
Person-years	268,473	192,354	36,182	23,796	13,639	2,502
No. of events	961	558	147	126	102	28
Events/100,000 person-years	358	290	406	529	748	1,119
Comorbidities						
Myocardial infarction	2,080 (3.1)	1,287 (2.7)	249 (2.9)	228 (3.9)	250 (6.5)	66 (7.7)
Stroke	1,620 (2.4)	1,085 (2.3)	190 (2.2)	143 (2.5)	158 (4.1)	44 (5.1)
Heart failure	5,132 (7.7)	2,778 (5.9)	656 (7.6)	513 (8.8)	872 (22.6)	313 (36.6)
Atrial fibrillation	6,817 (10.3)	4,144 (8.8)	881 (10.2)	700 (12.0)	861 (22.3)	231 (27.0)
Coronary heart disease	7,574 (11.4)	4,717 (10.0)	984 (11.4)	783 (13.4)	851 (22.1)	239 (27.9)
Hypertension	19,044 (28.7)	12,082 (25.6)	2,398 (27.7)	1,934 (33.2)	2,088 (54.2)	542 (63.3)
Peripheral artery disease	2,044 (3.1)	1,336 (2.8)	243 (2.8)	204 (3.5)	208 (5.4)	53 (6.2)
Diabetes mellitus type 2	6,135 (9.2)	3,946 (8.4)	744 (8.6)	576 (9.9)	690 (17.9)	179 (20.9)
DVT	1,050 (1.6)	673 (1.4)	138 (1.6)	143 (2.5)	66 (1.7)	30 (3.5)
Pulmonary embolism	831 (1.3)	462 (1.0)	123 (1.4)	108 (1.9)	101 (2.6)	37 (4.3)
Any cardiovascular disease^a^	24,919 (37.5)	16,031 (33.9)	3,112 (36.0)	2,450 (42.1)	2,650 (68.8)	676 (79.0)

a Any of the comorbidities presented in Table 2.

**Table 3:** Baseline COPD and Cardioprotective Medications

Medication Category	Total Study Population (N = 66,422)	No. of Exacerbations, Moderate/Severe				
		0/0 (n = 47,236)	1/0 (n = 8,652)	≥ 2/0 (n = 5,825)	0/1 (n = 3,853)	0/≥ 2 (n = 856)
COPD medication						
ICS	3,139 (4.7)	2,360 (5.0)	467 (5.4)	215 (3.7)	89 (2.3)	8 (0.9)
LABA	1,226 (1.8)	982 (2.1)	128 (1.5)	80 (1.4)	34 (0.9)	2 (0.2)
LAMA	8,710 (13.1)	6,881 (14.6)	935 (10.8)	456 (7.8)	407 (10.6)	31 (3.6)
SABA or SAMA	2,885 (4.3)	2,150 (4.6)	469 (5.4)	145 (2.5)	116 (3.0)	5 (0.6)
ICS/LABA	8,306 (12.5)	5,832 (12.3)	1,304 (15.1)	817 (14.0)	295 (7.7)	58 (6.8)
ICS/LABA/LAMA	20,017 (30.1)	10,871 (23.0)	3,369 (38.9)	3,003 (51.6)	2,111 (54.8)	663 (77.5)
ICS/LAMA	1,894 (2.9)	1,327 (2.8)	293 (3.4)	179 (3.1)	78 (2.0)	17 (2.0)
LABA/LAMA	5,086 (7.7)	3,628 (7.7)	654 (7.6)	390 (6.7)	357 (9.3)	57 (6.7)
No inhalation treatment	15,159 (22.8)	13,205 (28.0)	1,033 (11.9)	540 (9.3)	366 (9.5)	15 (1.8)
Cardioprotective medication						
Anticoagulation	25,851 (38.9)	17,447 (36.9)	3,322 (38.4)	2,464 (42.3)	2,100 (54.5)	518 (60.5)
Antiarrhythmic	7,421 (11.2)	4,510 (9.5)	1,089 (12.6)	900 (15.5)	717 (18.6)	205 (23.9)
Diuretics	731 (1.1)	459 (1.0)	120 (1.4)	77 (1.3)	69 (1.8)	6 (0.7)
Beta-blocker	16,170 (24.3)	9,881 (20.9)	2,215 (25.6)	1,870 (32.1)	1,676 (43.5)	528 (61.7)
Calcium channel blocker	23,244 (35.0)	15,750 (33.3)	2,993 (34.6)	2,192 (37.6)	1,853 (48.1)	456 (53.3)
ACE/ARB	16,967 (25.5)	11,890 (25.2)	2,070 (23.9)	1,545 (26.5)	1,202 (31.2)	260 (30.4)
Statin	29,630 (44.6)	20,882 (44.2)	3,719 (43.0)	2,671 (45.9)	1,933 (50.2)	425 (49.6)
Diabetes	24,136 (36.3)	17,215 (36.4)	2,935 (33.9)	2,093 (35.9)	1,534 (39.8)	359 (41.9)
Any cardiac agent^a^	8,906 (13.4)	6,278 (13.3)	1,032 (11.9)	792 (13.6)	642 (16.7)	162 (18.9)
Any metabolic^a^	46,460 (69.9)	32,149 (68.1)	5,999 (69.3)	4,423 (75.9)	3,135 (81.4)	754 (88.1)
Any cardiometabolic^a^	26,404 (39.8)	18,791 (39.8)	3,198 (37.0)	2,319 (39.8)	1,693 (43.9)	403 (47.1)

All combination therapies were fixed single inhaler combination therapies. ACE = angiotensin-converting enzyme inhibitor; ARB = angiotensin receptor blocker; ICS = inhaled corticosteroid; LABA = long-acting beta-2 agonist; LAMA = long-acting antimuscarinic agent; SABA = short-acting beta-2 agonist; SAMA = short-acting antimuscarinic agent.

a See e-Table 2 for definitions.

### Outcome of MI

For the outcome of MI, patients were followed for a median of 3.94 years (interquartile range, 2.26-5.60), for a total of 265,849 patient-years. There were 2,260 cases of MI during the follow-up; of these, 19.9% (n = 450) were out-of-hospital fatal events. In the crude model, the number of AEs of COPD at baseline increased the risk of MI in a gradual fashion (e-Table 3). The observed pattern was only slightly attenuated when adjusted for covariates. The adjusted SHR of MI in patients with one moderate exacerbation was not significantly elevated (1.10; 95% CI, 0.97-1.24); however, for patients with two or more moderate exacerbations without severe exacerbations, the SHR of MI was elevated (1.57; 95% CI, 1.38-1.78). For the group with one severe exacerbation, the SHR of MI was 1.47 (95% CI, 1.26-1.71); for the group with two or more severe exacerbations, the SHR was 1.82 (95% CI, 1.36-2.44) (Fig 3). The cumulative incidence function curves for MI are displayed in e-Figure 1.

### Outcome of PE

For PE, the median follow-up time was 3.72 years (interquartile range, 2.32-5.62), for a total of 268,473 patient-years. There were 961 cases of PE during the follow-up, of which 5.4% (n = 52) were out-of-hospital fatal events. In both the crude and adjusted model, the risk of PE was elevated in all exacerbation history strata compared with the reference group with zero exacerbations (e-Tables 3, 4; Fig 4). The adjusted SHR ranged from 1.33 (95% CI, 1.11-1.60) in patients with one moderate exacerbation without severe exacerbations to an adjusted SHR of 2.62 (95% CI, 1.77-3.89) in the group with two or more severe exacerbations (Fig 4). The cumulative incidence function curve for PE is displayed in e-Figure 2.

Both model findings were supported by similar results in the sensitivity analysis excluding patients who never smoked and the sleep apnea sensitivity analysis (e-Tables 5, 6). Of the 30% of patients with recorded data on the sleep apnea variable, 7.2% had a sleep apnea diagnosis.

In our time-restricted sensitivity analysis, the association between the exposure categories and the main outcomes of MI and PE were the strongest in the first year of follow-up. The results are presented in Tables 4 and 5. The associated risk increases diminished over time but were still significant and a bit more pronounced after 5 years compared with the maximum follow-up time of 9 years.

**Table 4:** Adjusted SHRs for Myocardial Infarction on Baseline Exacerbation History

Exacerbation History	Adjusted^a^ SHR (95% CI) for Myocardial Infarction		
Follow-up time maximum	1 y	2 y	5 y
0 exacerbations	Ref	Ref	Ref
1 moderate, 0 severe	1.28 (1.01-1.63)	1.12 (0.93-1.35)	1.13 (0.99-1.29)
≥ 2 moderate, 0 severe	1.80 (1.41-2.29)	1.77 (1.48-2.11)	1.61 (1.40-1.84)
1 severe	1.68 (1.27-2.21)	1.68 (1.37-2.05)	1.51 (1.29-1.77)
≥ 2 severe	2.88 (1.88-4.42)	2.30 (1.62-3.27)	1.87 (1.38-2.52)
Event rate per 100,000 patient-years	941	923	870

Ref = reference; SHR = subdistribution hazard ratio.

a Adjusted for sex, age, BMI, smoking status, FEV_1_ % predicted, presence of baseline cardiovascular comorbidity, cardioprotective medications, and socioeconomic factors.

**Table 5:** Adjusted SHRs for Pulmonary Embolism on Baseline Exacerbation History

Exacerbation History	Adjusted^a^ SHR (95% CI) for Pulmonary Embolism		
Restricted maximum follow-up time	1 y	2 y	5 y
0 exacerbations	Ref	Ref	Ref
1 moderate, 0 severe	1.59 (1.07-2.34)	1.49 (1.13-1.98)	1.34 (1.10-1.63)
≥ 2 moderate, 0 severe	2.76 (1.91-3.98)	1.89 (1.40-2.55)	1.60 (1.30-1.98)
1 severe	3.12 (2.10-4.63)	2.58 (1.89-3.50)	2.11 (1.67-2.66)
≥ 2 severe	4.54 (2.45-8.44)	3.71 (2.24-6.15)	2.63 (1.73-3.99)
Event rate per 100,000 patient-years	364	340	355

Ref = reference; SHR = subdistribution hazard ratio.

a Adjusted for sex, age, BMI, smoking status, FEV_1_ % predicted, presence of baseline cardiovascular comorbidity, cardioprotective medications, and socioeconomic factors.

## Discussion

To our knowledge, this is the first study to investigate the association across both number and severity of exacerbations among patients with COPD with the risk of MI and PE in a large, real-life cohort with disease-specific register data. We found that an exacerbation history with more frequent and/or severe exacerbations was independently associated with increasing risk of future MI and PE during a median of almost 4 years of follow-up, in total approximately 265,000 patient-years. In our time-restricted sensitivity analyses, the associated risk increase was most pronounced the first year of follow-up and then diminished slightly over time.

The current GOLD report^16^ recommends evaluating the number and severity of exacerbations during the last 12 months as an estimate of the risk of future exacerbations. In this clinically representative COPD cohort, a detailed exacerbation history could indeed independently identify patients at differential increased risk for cardiovascular and pulmonary embolic events.

Although long-term cardiovascular risk based on exacerbation history in the prior year has not been previously studied, increased cardiovascular risk during and shortly after an exacerbation of COPD has been previously reported. In a UK cohort based on medical records, the authors reported a 2.27-fold higher risk of MI in the 5 days after the start of pharmacologic treatment for an exacerbation, and patients with MI had higher rates of exacerbations.^15^ A post hoc analysis of the Study to Understand Mortality and Morbidity in COPD (SUMMIT) trial found a near 10 times increased risk of cardiovascular disease events (stroke, transient ischemic attacks, and unstable angina) in the 30 days after hospitalization for an AECOPD.^13^ Similarly, a post hoc analysis of the Informing the Pathway of COPD Treatment (IMPACT) trial found a higher risk of cardiovascular disease events (new or worsened cardiac arrhythmia, cardiac failure, ischemic heart disease, hypertension, and central nervous system hemorrhages and cerebrovascular conditions) during and in the 30 days after the resolution of an exacerbation.^11^ Furthermore, in a self-controlled case series studying different time periods after the incidence of an AE of COPD, the authors found a risk increase for both MI and ischemic stroke in the 1 to 91 days after the onset of an acute exacerbation.^10^

Previous studies have also identified a substantial prevalence of PE during AEs of COPD in hospitalized patients within 48 h after admittance for AE of COPD, with pooled prevalence estimates in meta-analyses ranging from 11.0% to 19.9%.^27^, ^28^, ^29^ PE is associated with increased mortality, and in patients with COPD the risk of death when diagnosed with PE might be twice as high compared with patients without COPD.^9^^,^^30^^,^^31^ Further larger cohort studies are needed to establish potential new screening algorithms for PE in the setting of hospitalization for AE of COPD.^32^ Our study adds to this previous knowledge by highlighting an important longer-term longitudinal association between AEs of COPD and MI and PE, and the utility of a detailed exacerbation history in the assessment of these life-threatening events.

Risk reduction of exacerbations is a key objective of COPD management, and our results further emphasize its importance for cardiovascular risk. Interestingly, in two large randomized controlled trials evaluating the effects of LABA/LAMA/ICS vs LABA/LAMA on exacerbation risk, a reduction of all-cause mortality, and cardiovascular mortality specifically, was demonstrated.^33^^,^^34^ The potential risk reduction of cardiovascular and thromboembolic events by antiplatelet therapy in frequent exacerbators is promising, but remains to be studied in randomized controlled trials.

The results also serve to further emphasize the importance of cardiovascular and other comorbidity in COPD, which is often unassessed and undiagnosed.^28^^,^^35^ It is important to carefully consider differential diagnoses of a suspected COPD exacerbation in patients with COPD presenting with worsening dyspnea in the acute care setting, and this includes particularly MI and PE.^27^^,^^28^^,^^32^^,^^36^^,^^37^

Sleep apnea is an important comorbidity in COPD and a potential confounder between COPD exacerbations and cardiovascular disease. Although we excluded it from the main model due to the large portion of missing data, our sensitivity analysis, where sleep apnea was included as a covariate in our competing risk regression model, indicated that the risk of the main outcomes was of similar magnitude even when adjusting for sleep apnea.

Two main strengths of this study are the size of the study cohort, with an estimated observation time of approximately 265,000 person-years, and the real-life setting reflecting clinical COPD practice in Sweden. Additionally, the SNAR provides a comprehensive and detailed assessment of demographic and clinical characteristics of the patients with COPD studied. A further strength is the linkage with population-based health registries through the unique national personal identity number.^38^^,^^39^ These registers provided 100% complete data for the prescription of COPD treatments and cardioprotective medications, hospitalization, and mortality for the patients in our study. By using the latest registered FEV_1_ value for cohort selection, we ensured complete data on this important variable which has previously been linked to cardiovascular disease outcomes and increased mortality in patients with COPD.^40^^,^^41^

One study limitation is that we used a definition of moderate exacerbations which did not include exacerbations treated with antibiotics alone, which may lead to an underestimation of moderate exacerbations. However, this bias is likely to be small and a prescription-based definition only using oral corticosteroids has been shown to be a robust definition of moderate COPD exacerbations.^21^ Additionally, MIs can have different etiologies, the most common being plaque rupture and thrombosis causing acute coronary artery occlusion (type 1 infarction). However, it can also be caused by a mismatch in blood oxygen supply and demand in the heart (type 2 infarction), a mechanism which could be important and potentially more common in patients with COPD hospitalized for AE of COPD, who often are affected by hypoxemia.^42^ However, a limitation of ICD-10 codes for MI is that they do not differentiate between these types.

Furthermore, the registered diagnosis in NCDR collected from death certificates is most often based on clinical diagnosis because clinical autopsies are rarely performed in Sweden unless the individual died unnaturally or under unclear circumstances calling for a forensic autopsy. Although this procedure is similar to other countries, death certificates based on clinical diagnosis nonetheless represent a possible source of inaccuracy and bias compared with cause of death-based autopsies. Likewise, the SNAR diagnosis of COPD relies on a physician-registered primary diagnosis of COPD. It is not possible to validate an individual COPD diagnosis within the SNAR, due to the nature of the real-life register data, and some patients might therefore be misdiagnosed patients with asthma (however asthma has its own physician-defined diagnosis variable within the SNAR). Fixed airflow obstruction in patients who never smoked could be misclassified asthma. However, our sensitivity analysis excluding patients who never smoked yielded results that were reassuring to our main analysis.

In summary, we found that patients who had severe or frequent exacerbations had a long-term increased risk of MI and PE. We conclude that the stratification of patients with COPD based on their exacerbation history in the last year can be a valuable assessment of the future risk of MI and PE. A detailed assessment of cardiovascular comorbidity is warranted in patients with COPD and frequent or severe exacerbations, in a stable phase but also during worsening dyspnea. Future studies should evaluate primary cardiovascular risk reduction strategies in frequent exacerbators.

## Funding/Support

L. E. G. W. V. received support from The Family Kamprad Foundation [Grant 20190024], The Swedish Heart and Lung Foundation [Grant 20200150], the Swedish government and country council ALF grant [Grant ALFGBG-824371], and the Swedish Society of Respiratory Medicine (SLMF). C. S. received support from the Swedish Heart and Lung Foundation [Grant 20200549].

## Financial/Nonfinancial Disclosures

The authors have reported to *CHEST* the following: L. E. G. W. V. reports relationships with GSK, AstraZeneca, Boehringer Ingelheim Pharmaceuticals Inc, Novartis, Chiesi, Resmed, and Pulmonx that includes speaking and lecture fees. C. S. reports relationships with AstraZeneca, Chiesi, Boehringer Ingelheim, and TEva that includes consulting or advisory and speaking and lecture fees. A. L. reports relationships with Boehringer Ingelheim, Novartis, GlaxoSmithKline, and AstraZeneca that includes consulting or advisory and speaking and lecture fees. F. N. was previously an employee of AstraZeneca until 2019 and owns stock in the company. None declared (O. W.).

## Supplementary Data

e-Online Data

e-Online Data

e-Online Data

e-Online Data
