# Supplementary material for: Exacerbation History and Risk of Myocardial Infarction and Pulmonary Embolism in COPD
Source: Chest. 2024 Jul 31;166(6):1347–59. doi: 10.1016/j.chest.2024.07.150 (PMC11638550; doi:10.1016/j.chest.2024.07.150)
Supplement: e-Online Data [file mmc2.docx]

*E-Figure 2.* CIF-plot of cumulative incidence functions for outcome of pulmonary embolism with death from other causes as a competing risk, 66422 Patients with COPD divided by baseline exacerbation history
