# Supplementary material for: Exacerbation History and Risk of Myocardial Infarction and Pulmonary Embolism in COPD
Source: Chest. 2024 Jul 31;166(6):1347–59. doi: 10.1016/j.chest.2024.07.150 (PMC11638550; doi:10.1016/j.chest.2024.07.150)
Supplement: e-Online Data [file mmc3.docx]

# Supplementary

## LIST OF ATC CODES FOR INHALER THERAPY

## **Table E1:**Drug therapy classes and ATC codes for respiratory inhaler therapy used in this study from the Swedish Prescribed Drug Register.

| **Drug therapy class** | **ATC codes for respiratory inhaler therapy** |
| --- | --- |
| SABA and/or SAMA | - R03AC02, Salbutamol - R02AC03, Terbutalin - R03BB01, Ipratropiumbromid - R03AL02, Salbutamol and ipratropiumbromid |
| LAMA | - R03BB01, Ipratropiumbromid - R03BB04, Tiotropiumbromid - R03BB05, Aclidiniumbromid - R03BB06, Glycopyrroniumbromid - R03BB07, Umeclidiniumbromid |
| LABA | - R03AC12, Salmeterol - R03AC13, Formoterol - R03AC18, Indacaterol - R03AC19, Olodaterol |
| ICS | - R03BA01, Beclometason - R03BA02, Budesonide - R03BA04, Betametason - R03BA05, Fluticason - R03BA07, Mometason - R03BA08, Ciklesonid |
| LABA/LAMA | - R03AL03, Vilanterol and Umeclidiniumbromid - R03AL04, Indacaterol and Glycopyrroniumbromid - R03AL05, Formoterol and Aclidiniumbromid - R03AL06, Olodaterol and Tiotropiumbromid - R03AL07, Formoterol and Glycopyrroniumbromid - R03AL10, Formoterol and Tiotropium |
| LABA/ICS | - R03AK06, Salmeterol and Fluticason - R03AK07, Formoterol and Budesonid - R03AK08, Formoterol and Beclometason - R03AK09, Formoterol and Mometason - R03AK10, Vilanterol and Fluticasonfuroat - R03AK11, Formoterol and Fluticason - R03AK12, Salmeterol and Budesonid - R03AK13, Salmeterol and Budesonid - R03AK14, Indakaterol and Mometason |
| LABA/LAMA/ICS | - R03AL08, Vilanterol, umeklidiniumbromid and flutikasonfuroat - R03AL09, Formoterol, Glycopyrroniumbromid and Beclometason - R03AL11, Formoterol, Glykopyrroniumbromid, Budesonide - R03AL12, Indakaterol, Glukopyrronium, Mometason |

**Table E2:** Drug therapy classes and ATC codes for cardioprotective medications used in this study from the Swedish Prescribed Drug Register.

| **Drug therapy class** | **ATC code groups** |
| --- | --- |
| Anticoagulation | B01 |
| Anti-arrhythmic | C01 |
| Diuretics | C03 |
| Betablocker | C07 |
| Calcium channel-blocker | C08 |
| ACE/ARB | C09 |
| Statin | C10 |
| Diabetes | A10 |
| Any cardiac drug | B01  C01  C02  C03  C07  C08  C09 |
| Any Metabolic drug | C10  A10 |
| Any Cardiometabolic drug | B01  C01  C02  C03  C07  C08  C09  A10 |

**Table E3.** Crude subdistribution hazard ratios (SHRs) from competing risk regression analysis of risk of myocardial infarction (left) and pulmonary embolism (right) side-by-side comparison in 66 422 Swedish COPD patients stratified by baseline exacerbation history

| **Exacerbation history** | **Main results crude SHR 95% CI for myocardial infarction** | **Main results crude SHR 95% CI for pulmonary embolism** |  |
| --- | --- | --- | --- |
| 0 Exacerbations | Ref | Ref |  |
| 1 Moderate | 1.08 (0.95-1.22) | 1.40 (1.17-1.68) |  |
| >2 Moderate | 1.63 (1.44-1.85) | 1.83 (1.50-2.22) |  |
| 1 Severe | 1.92 (1.65-2.23) | 2.59 (2.09-3.19) |  |
| 2+ Severe | 2.58 (1.94-3.44) | 3.87 (2.65-5.67) |  |

**Table E4.** Adjusted subdistribution hazard ratios (SHRs) from competing risk regression analysis of risk of myocardial infarction (left) and pulmonary embolism (right) side-by-side comparison in 66 422 Swedish COPD patients stratified by baseline exacerbation history

| **Exacerbation history** | **Main results adjusted SHR 95% CI for myocardial infarction** | **Main results adjusted SHR 95% CI for pulmonary embolism** |  |
| --- | --- | --- | --- |
| 0 Exacerbations | Ref | Ref |  |
| 1 Moderate | 1.10 (0.97-1.24) | 1.33 (1.11-1.60) |  |
| >2 Moderate | 1.57 (1.38-1.78) | 1.60 (1.31-1.94) |  |
| 1 Severe | 1.47 (1.26-1.71) | 1.99 (1.59- 2.49) |  |
| 2+ Severe | 1.82 (1.36-2.44) | 2.62 (1.77-3.89) |  |

1 *Adjusted for sex, age, BMI, smoking status, FEV_1_ % predicted, the presence of baseline cardiovascular comorbidity and cardioprotective medications, income and educational level*

**Table E5** Adjusted^1^ subdistribution hazard ratios (SHRs) for myocardial infarction and pulmonary embolism depending on baseline exacerbation history, from competing risk regression analysis for 54 726 Swedish patients diagnosed with COPD who previously or currently smoked, registered in the Swedish National Airway Register January 2014 - June 2022 and followed for up to 9 years.

| **Exacerbation history** | **Adjusted^1^ SHR 95% CI for myocardial infarction** | **Adjusted^1^ SHR 95% CI for Pulmonary Embolism** |
| --- | --- | --- |
| 0 Exacerbations | Ref | Ref |
| 1 Moderate | 1.08 (0.94-1.24) | 1.33 (1.08-1.63) |
| >2 Moderate | 1.57 (1.37-1.80) | 1.62 (1.30-2.02) |
| 1 Severe | 1.43 (1.21-1.69) | 2.14 (1.68 -2.73) |
| 2+ Severe | 1.59 (1.14-2.20) | 2.89 (1.91-4.38) |

1 *Adjusted for sex, age, BMI, FEV_1_ % predicted, the presence of baseline cardiovascular comorbidity and cardioprotective medications, income and educational level.*

**Table E6** Adjusted^1^ subdistribution hazard ratios (SHRs) for myocardial infarction and pulmonary embolism depending on baseline exacerbation history, from competing risk regression analysis for 66 422 Swedish patients diagnosed with COPD, with added adjustment for sleep apnea with missing as a factor level, registered in the Swedish National Airway Register January 2014 - June 2022 and followed for up to 9 years.

| **Exacerbation history** | **Adjusted^1^ SHR 95% CI for myocardial infarction** | **Adjusted^1^ SHR 95% CI for Pulmonary Embolism** |
| --- | --- | --- |
| 0 Exacerbations | Ref | Ref |
| 1 Moderate | 1.11 (0.98-1.27) | 1.24 (1.02-1.51) |
| >2 Moderate | 1.60 (1.41-1.83) | 1.64 (1.33-2.02) |
| 1 Severe | 1.45 (1.23-1.70) | 2.05 (1.62-2.58) |
| 2+ Severe | 1.64 (1.19-2.27) | 2.84 (1.90-4.25) |

1 *Adjusted for sex, age, BMI, smoking status, FEV1 % predicted, the presence of baseline cardiovascular comorbidity, cardioprotective medications and sleep apnea*
